# Reevaluating heparin reversal in managing intraprocedural ruptures: A call for tailored approaches in endovascular treatment of intracranial aneurysms

**DOI:** 10.1016/j.bas.2024.102921

**Published:** 2024-08-14

**Authors:** Ayush Anand, Muhammed Shabil, Nitin Kumar Bansal, Sanjit Sah, Olabisi Oluwagbemiga Ogunleye

**Affiliations:** BP Koirala Institute of Health Sciences, Dharan, Nepal; Center for Global Health Research, Saveetha Medical College and Hospital, Saveetha Institute of Medical and Technical Sciences, Saveetha University, Chennai, India; Evidence for Policy and Learning, Global Center for Evidence Synthesis, Chandigarh, India; Department of General Medicine, Graphic Era (Deemed to Be University) Clement Town, Dehradun, 248002, India; Department of Paediatrics, Dr. D. Y. Patil Medical College, Hospital and Research Centre, Dr. D. Y. Patil Vidyapeeth, Pune, 411018, Maharashtra, India; Department of Public Health Dentistry, Dr. D.Y. Patil Dental College and Hospital, Dr. D.Y. Patil Vidyapeeth, Pune, 411018, Maharashtra, India; Neurosurgery, Department of Surgery, Abubakar Tafawa Balewa University, Bauchi, Nigeria

Intraprocedural rupture (IPR) during endovascular treatment (EVT) for intracranial aneurysms is a critical complication that can lead to devastating outcomes. A recent study by Hirai et al. brings to light essential insights regarding the safety and efficacy of various management strategies for IPR, focusing on the implications of heparin reversal (Hirai et al., 2024). The study underscores the complexity of managing IPR, particularly the role of heparin reversal. Traditionally, heparin reversal is employed to mitigate bleeding complications during IPR by neutralizing anticoagulation (Ihn et al., 2018). However, the findings suggest that while heparin reversal might aid in hemostasis, it is significantly associated with developing ischemic complications. This dichotomy presents a clinical conundrum: balancing the need for immediate bleeding control with the risk of thromboembolic events.

While the study provides valuable insights, it also has several limitations that must be acknowledged. First, the study's retrospective nature introduces inherent biases related to data collection and patient selection. Prospective, randomized studies are needed to validate these findings. Second, although the study involves multiple centers, the relatively small number of IPR cases (74 out of 3269) limits the generalizability of the findings. More extensive studies are required to confirm these results. Third, the study included patients with both ruptured and unruptured aneurysms, which may introduce variability in outcomes. A more homogeneous patient population could provide more precise insights. Fourth, variations in EVT techniques and IPR management protocols across centers might affect the study's findings. Standardized treatment protocols would help in obtaining more consistent data. Fifth, the study relies on qualitative imaging assessments and clinical outcomes, which might lack the precision of quantitative measures. Advanced imaging techniques and standardized outcome metrics could enhance the accuracy of the results.

Several steps can be taken to address these limitations and build on the findings of Hirai et al. First, conducting prospective, multicenter studies with larger patient populations will help validate the current findings and reduce biases associated with retrospective analyses. Second, developing and implementing standardized protocols for EVT and IPR management across centers will ensure consistency in treatment approaches and outcomes. Third, focusing on more homogeneous patient populations, such as separate studies for ruptured and unruptured aneurysms, will provide clearer insights into the specific challenges and outcomes associated with each group. Fourth, utilizing advanced imaging modalities, such as high-resolution MRI and quantitative CT, will provide more precise assessments of hemorrhagic and ischemic changes, leading to better outcome measurements. Fifth, emphasizing the need for individualized treatment plans based on patient-specific factors, such as age, aneurysm characteristics, and comorbidities, will improve the efficacy of IPR management.

The study by Hirai et al. is a significant contribution to neuroendovascular surgery, providing crucial insights into the management of IPR during EVT for intracranial aneurysms. The findings challenge the conventional use of heparin reversal and highlight the need for tailored approaches to improve patient outcomes. By addressing the study's limitations through prospective research, standardized protocols, and advanced imaging, the neurosurgical community can develop more effective and individualized strategies for managing this devastating complication. As we continue refining our techniques and approaches, the ultimate goal remains to enhance patient safety and optimize clinical outcomes in treating intracranial aneurysms.

## Ethical approval

Not applicable.

## Consent to participate

Not applicable.

## Consent for publication

Not applicable.

## Funding

This research received no specific grant from any funding agency in the public, commercial, or not-for-profit sectors.

## Availability of data and materials

Not applicable.

## Author contribution

Ayush Anand = Writing-original draft and Writing-review and editing.

Muhammed Shabil = Writing-original draft and Writing-review and editing.

Nitin Kumar Bansal = Writing-original draft and Writing-review and editing.

Sanjit Sah = Writing-original draft and Writing-review and editing.

## Declaration of competing interest

The Author(s) declare(s) that there is no conflict of interest.

## References

[bib1] Hirai S., Hanazawa R., Yoshimura M. (2024). Safety and efficacy of management for intraprocedural rupture during endovascular treatment for intracranial aneurysms. Neurosurgery.

[bib2] Ihn Y.K., Shin S.H., Baik S.K., Choi I.S. (2018). Complications of endovascular treatment for intracranial aneurysms: management and prevention. Intervent Neuroradiol..

